# Optical Properties of Polypropylene upon Recycling

**DOI:** 10.1155/2013/354093

**Published:** 2013-10-28

**Authors:** Felice De Santis, Roberto Pantani

**Affiliations:** Department of Industrial Engineering, University of Salerno, Via Giovanni Paolo II, 132-84084 Fisciano, Italy

## Abstract

In the last few years there has been an increasing interest in the possibility of recycling polymeric materials, using physical recycling. However, is it well known that polymers experience a depletion of all the properties upon recycling. These effects have been widely characterized in the literature for what concerns the mechanical or rheological properties. The changes of optical properties after recycling have been much less studied, even if, especially in food packaging, optical characteristics (above all the opacity) are of extreme importance, and thus it is quite significant to assess the effect of recycling on these properties. In this work, the influence of recycling steps on the opacity of films of a commercial grade of isotactic polypropylene (i-PP) was studied. The material was extruded several times to mimic the effect of recycling procedures. After extrusion, films were obtained by cooling samples of material at different cooling rates. The opacity of the obtained films was then measured and related to their crystallinity and morphology. It was found that opacity generally increases on increasing the amount of **α** phase and for the same amount of **α** phase on increasing the size of the spherulites.

## 1. Introduction

Plastic films for packaging are showing constant development due to their good quality/prices ratio, lightness, and flexibility in adapting to a broad range of packaging types. Optical clarity is another property which is critical for the success of plastics for food packaging, pallet wrap, or other related applications.

Among other plastics, isotactic polypropylene (i-PP) is a commodity polymer produced and used in large quantities in packaging. The main reasons for the success of i-PP are its quite good price/performance ratio, its excellent mechanical properties, and suitable optical characteristics [1]. The huge consumption of this polymer makes its recycling strategically very important for the environmental policy of industry [2].

Obviously, recycling induces deep changes in the material: recycled i-PP exhibits lower viscosity [3], faster crystallization rate, and higher crystallinity and equilibrium melting temperature than those measured for virgin i-PP [4]; elastic modulus and yield stress increase with the number of recycling steps [5]; elongation at break and fracture toughness decrease. Moreover, volatile organic compounds (VOCs) emitted during multiple melt reprocessing increase with increasing processing cycle [6] and could be correlated to structural/rheological changes of polypropylene [7].

The effect of recycling on optical properties has been marginally analyzed in the literature [8]. The optical transparency in semicrystalline polymers is mainly related to the crystallinity and to the surface properties [9]. Surface scattering is one of the major reasons for the loss of optical transparency [10]. Crystallinity affects optical transparency because of the scattering taking place when light passes from amorphous to crystalline regions: spherulites in i-PP are much larger than the wavelength of visible light (0.4–0.7 *μ*m), and the refractive index of crystalline regions is higher than that of amorphous regions; as light rays pass from amorphous to crystalline regions, they encounter the large spherulites, resulting in light scattering; as a result, optical transparency is lower, and haze is produced. Due to their noncrystalline structure, amorphous materials have better optical transparency than semicrystalline materials, and a decrease in crystallinity of a semicrystalline polymer enhances the clarity [11]. However, excessive reductions in crystallinity can result in unacceptable reductions in strength, stiffness, and resistance to softening, so a compromise must be reached that is appropriate for the application.

In this work, the influence of recycling steps on the opacity of films of a commercial grade of isotactic polypropylene (i-PP) was studied. The material was extruded several times to mimic the effect of recycling procedures. After extrusion, films were obtained by cooling samples of material at different cooling rates. The opacity of the obtained films was then measured and related to their crystallinity and morphology.

## 2. Materials and Methods

The i-PP homopolymer Moplen HP450J produced by Lyondell-Basell was used in the experiments. Moplen HP540J is a nucleated homopolymer for extrusion and thermoforming applications. Moplen HP540J exhibits a good stiffness and optical transparency and is thus adopted for fruit baskets, trays, transparent drinking cups, and containers.

The selected process was extrusion, and materials subjected to zero, five, and ten steps of recycling were analyzed. The processing conditions adopted during extrusion are reported in Table 1.

Each material was then formed as 150 *μ*m thick films at three different cooling rates, by using a device [12, 13] able to impose cooling conditions in the range of interest for polymer processing, namely, from 0.1 K/s to more than 100 K/s. The cooling rates imposed by the device are not constant with temperature, with the driving force being essentially dictated by the difference between sample and cooling media temperatures. For this reason, the cooling rate measured at 70°C is usually chosen as a reference to identify a particular cooling history, as suggested in the literature [14] for i-PP. In this work, the films were solidified at cooling rates (measured at 70°C) of the order of 0.1, 10, and 100 K/s (the exact values are reported in Table 2) in order to assess the effect of different crystallinity degrees on the optical properties. According to the procedure adopted, the film is solidified between two thin glasses, and thus the surface finish is the same for all the samples. On each of the obtained films measurements of crystallinity degrees, birefringence, and opacity were performed.

The opacity of the films was measured by using a KonicaMinolta X-Rite SP60 Series Spectrophotometer. Following the ASTM Book of Standards E 284 “Standard Terminology of Appearance” [15], opacity is the ability of a thin film or sheet of material, such as paint or paper, to hide a surface behind and in contact with it, expressed as the ratio of the reflectance factor *R*_*b*_ when the material is backed by a black surface to the reflectance factor *R*_*w*_ when it is backed by a white surface (usually having a reflectance factor of 0.89):

(1) $$\begin{array}{c} \text{opacity}=\frac{R_{b}}{R_{w}}100. \end{array}$$

The samples solidified under different cooling rates were analyzed by means of an M2000 Fourier transform infrared (FTIR) spectrometer by Midac Co., measuring the absorbance in the range 400–4000/cm wavenumbers.

Wide-angle X-ray scattering (WAXS) characterization was carried out by a D8-Advance Bruker-AXS diffractometer using Cu K*α* irradiation.

An optical polarizer microscope was adopted to measure the birefringence of the films by analyzing the optical retardation.

The samples were then etched in order to remove the amorphous portions, and optical microscopy was used to take images of the samples and measure the dimensions of the spherulites.

Optical images and birefringence measurements were taken by using a BX-41 Olympus polarized microscope equipped with a digital camera.

## 3. Results and Discussion

**Figure 1:**
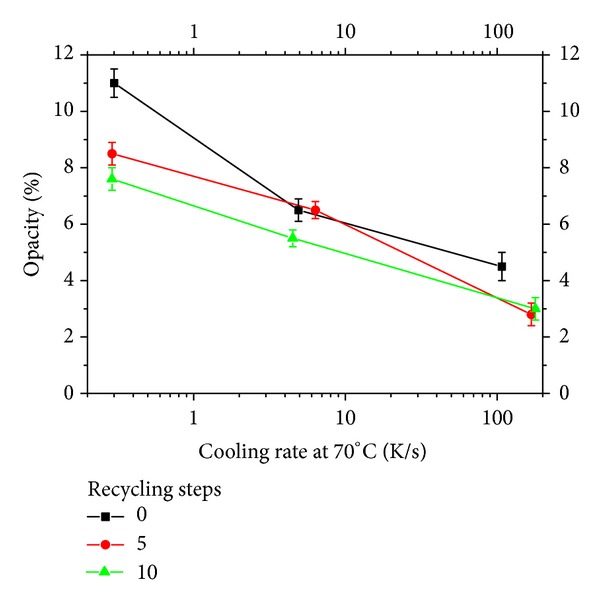
Effect of cooling rates and recycling steps on the opacity of the films.

The effect of cooling rates and recycling on the opacity of the films is reported in Figure 1. As a general result it can be noticed that opacity reduces on increasing the cooling rate. Somewhat surprisingly, opacity seems to decrease on increasing the steps of recycling, and this effect is more evident at high cooling rates. In order to investigate this phenomenon, a complete morphological characterization of the samples was carried out.

**Figure 2:**
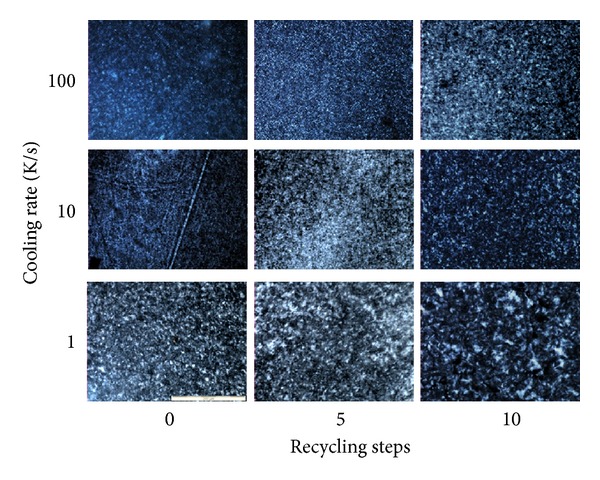
Optical images of the samples analyzed in this work: on the same row samples subjected to different recycling steps but solidified under cooling rates of the same order of magnitude are reported; on the same column samples having the same recycling history but solidified under different cooling rates are reported. All the images are on the same magnification scale; namely, the longest side is about 400 *μ*m.

The optical images of the etched samples are reported in Figure 2: on the same row, samples subjected to different recycling steps but solidified under cooling rates of the same order of magnitude are reported; on the same column samples having the same recycling history but solidified under different cooling rates are reported. All the images are on the same magnification scale. It can be noticed that on increasing the cooling rate, the final dimension of the crystalline structures (the diameter of the spherulites) reduces [16].

From the optical images, it was possible to estimate the average radius of the spherulites for some of the samples, as reported in Table 2. For the samples solidified at the highest cooling rates, it was not possible by optical images to identify clear structures to be measured.

**Figure 3:**
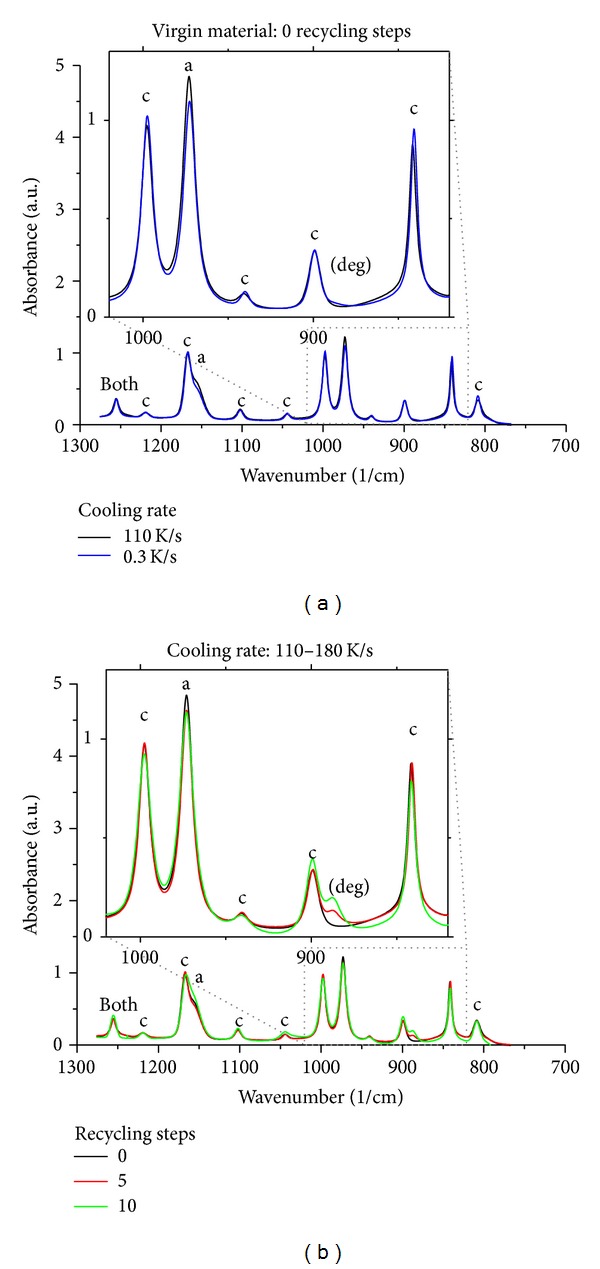
FTIR spectra collected on some of the samples analyzed in this work. In plot (a) spectra of virgin samples solidified at two cooling rates; in plot (b) spectra of samples undergone different recycling steps and solidified at the fastest applied cooling rate. The insets show a magnification of the plots.

In order to fully characterize the morphology of the samples, FTIR analysis was carried out. In Figure 3, the spectra in the region 1300–750/cm collected on some of the samples analyzed in this work are reported. In particular, in the left plot of Figure 3 the spectra of two samples solidified at two cooling rates (the lowest, 0.3 K/s, and the fastest, 110 K/s) are compared. For i-PP, several absorption bands of the crystalline and amorphous fraction have been identified [17], and the commonly adopted ones are highlighted in Figure 3. The most defined and isolated one is at 841/cm due to CH_2_ rocking and CH axial bending. Another band partially overlapping the first one is the band at 998/cm, due to CH_3_ equatorial rocking, C–CH_3_ stretching, CH, bending and CH_2_ twisting. All the mentioned bands are sensitive to the order of long helicoidal chains, and then they measure the contribution to order of *α* phase as well as of *β* phase and of mesomorphic structures. Thus crystallinity degree as measured by IR is an average crystallinity degree: it is not possible to discriminate between contribution of different phases. In plot (a) of Figure 3 it can be noticed that the peaks corresponding to the amorphous phase are slightly more pronounced, and conversely the peaks corresponding to the crystalline phase are slightly less pronounced, for the sample solidified at the highest cooling rate. The effect is more evident on zooming in a narrower region, as done in inset of the plot (a) of Figure 3. This indicates that, as expected, crystallinity slightly decreases on increasing cooling rate. The effect of recycling steps is analyzed in plot (b) of Figure 3, where samples undergoing different recycling steps and solidified at the fastest applied cooling rate (of the order of 100 K/s) are compared. The spectra look quite similar with some differences for the shoulder at 1158/cm (assigned to the amorphous phase [18]) which is more pronounced for the sample which underwent 10 steps of recycling and for the peak at 888/cm which increases on increasing the recycling steps. This latter peak is attributed to the external vinylidene groups, which are formed by disproportionation between free radicals formed by rupture of the polymer backbone and are an index of thermal oxidation [19]. The increase of the peak at 888/cm is a clear indication of thermal degradation of the material on increasing the recycling steps.

For a quantitative determination of the crystallinity degree, the FTIR absorbance spectra were analyzed applying Lambert and Beer's law to selected peaks [20].

Considering a crystalline and an amorphous peak and assuming that the absorbance of both the amorphous and the crystalline phases does not depend on phase-content distribution, Lambert and Beer's law provides, respectively,

(2) $$\begin{array}{c} A_{\text{cr}}=a_{\text{cr}}\cdot S\cdot \chi ,\\ A_{\text{am}}=a_{\text{am}}\cdot S\cdot \left(1-\chi \right), \end{array}$$

where  *A*_cr_  and  *A*_am_  are the absorbancies;  *a*_cr_  and  *a*_am_  are the absorption coefficients of the crystalline and amorphous phases peak, respectively;  *χ*  is the crystallinity degree; *S* is the sample thickness.

The value of *χ* may be obtained by eliminating  *S*  from (2):

(3) $$\begin{array}{c} \chi =\frac{A_{\text{am}}}{\left[A_{\text{cr}}+\left(a_{\text{cr}}/a_{\text{am}}\right)A_{\text{am}}\right]}. \end{array}$$

The value of *χ* can be thus calculated from measurements of absorbance if the ratio of absorption coefficients is known. This parameter is normally estimated using an independent experimental technique. In the literature, the value of 0.58 is found for i-PP [21].

The band at 841/cm was chosen for crystalline phase and the band 973/cm for amorphous phase. Because the spectra are the weighted superposition of single absorption peaks, all the absorbencies were obtained by fitting the experimental spectra with a weighted combination of single peaks, adopting Gaussian/Lorentzian peak functions.

**Figure 4:**
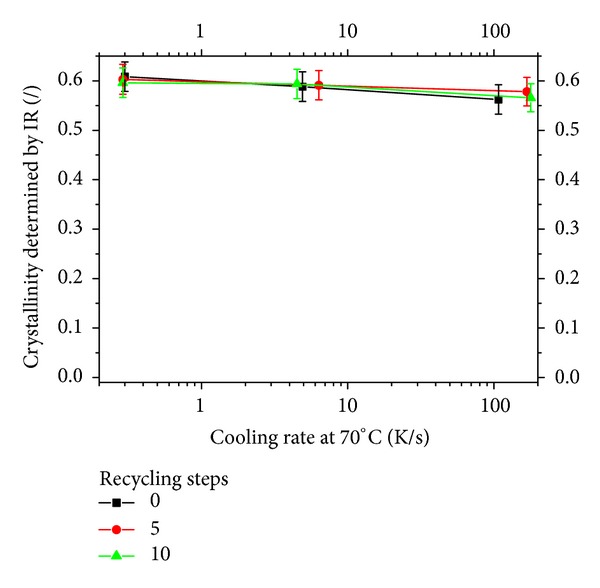
Overall crystallinity degree as assessed by IR spectroscopy.

The results are reported in Figure 4 and show that the overall crystallinity degree, as assessed by IR spectroscopy, only slightly decreases by effect of the cooling rate. This means that opacity, which is different from sample to sample as shown in Figure 1, is not determined by the overall crystallinity degree alone.

**Figure 5:**
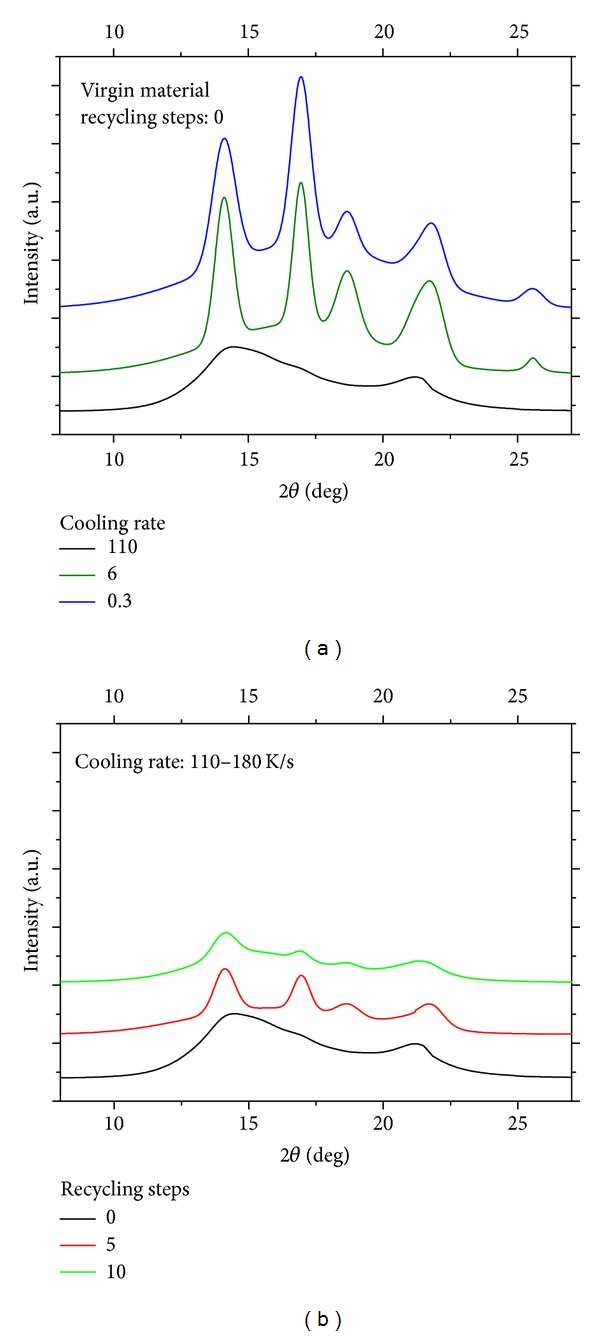
WAXS spectra of the samples of virgin material solidified at different cooling rates in the (a) plot and WAXS spectra of the samples solidified at the highest applied cooling rates in the (b) plot.

As mentioned above, FTIR analysis does not allow discriminating between different crystalline phases, and thus the crystallinity degree as measured by FTIR has to be considered as an overall value accounting for all existing crystalline phases. Thus in order to discriminate between different crystalline phases, the samples were analyzed using wide-angle X-ray scattering (WAXS). Plot (a) of Figure 5 shows the WAXS spectra of the samples of virgin material (0 recycling steps) solidified at different cooling rates. It can be noticed that, on increasing the cooling rate, the spectrum changes from that characteristic of the *α* phase to that characteristic of the mesomorphic or smectic phase. This is a result commonly found in the literature [12]. The effect of recycling steps on the samples solidified at the highest applied cooling rates is shown in plot (b) of Figure 5. Despite of the fast cooling rate, the sample subjected to 5 steps presents clear peaks characteristic of the *α* phase, indicating a faster crystallization kinetics with respect to the virgin material. This is probably due to a reduction of molecular weight (and thus to an increase of molecular mobility) induced by thermomechanical degradation [4]. The sample subjected to 10 steps of recycling presents an intermediate morphology between the virgin and the sample subjected to 5 steps: probably the increase of degradation slows to some extent the crystallization kinetics.

**Figure 6:**
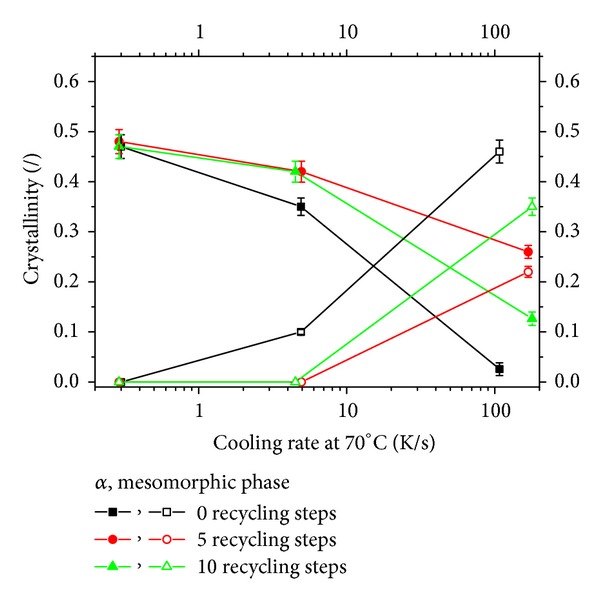
Content of *α* (solid symbol) and mesomorphic phase (open symbol) in the samples assessed by WAXS spectra deconvolution.

In order to reach a quantitative description of phase distribution inside the samples, the WAXS patterns were analyzed by a deconvolution procedure performed according to a scheme reported in the literature [12]. Results are given in Figure 6 and show that, on increasing the cooling rate, the amount of *α* phase reduces, whereas the amount of mesomorphic phase increases; on increasing the steps of recycling, the content of *α* phase generally increases; however, the largest amount of *α* phase is found in the samples undergone 5 steps of recycling, so that it can be concluded that the increase of *α* phase crystallization kinetics with recycling steps is not monotonous. This behavior has already been reported in the literature [4]. It is interesting to notice that, on summing up the contents of *α* and mesomorphic phases, an about constant value is reached for the samples and all the cooling rates, thus confirming the results of IR analysis (Figure 4). The differences in the total crystallinity content measured by the two techniques could be due to the ratio of absorptivities: probably the value of 0.58 found in the literature is not appropriate for this specific material i-PP; on using 0.62 both techniques give similar values.

**Figure 7:**
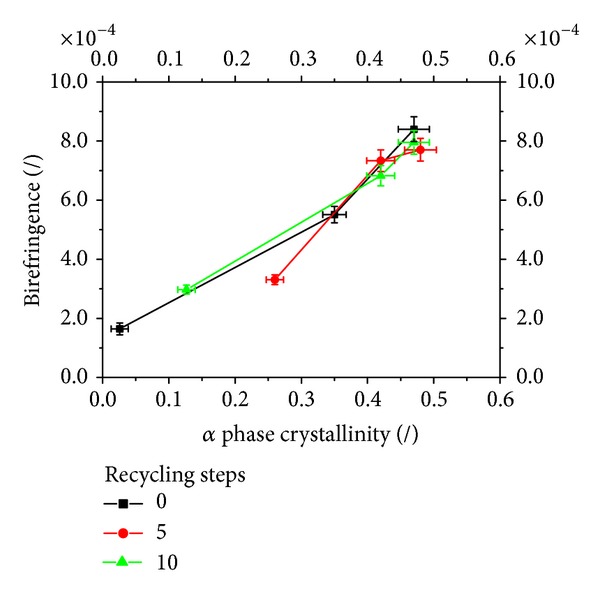
Measured birefringence of all the samples reported versus the amount of *α* phase of each film.

Birefringence is another important optical property of a polymeric film. It is the optical phenomenon in which a polymer sample exhibits different refractive indexes for light with plane polarization in two perpendicular directions. In unoriented polymer samples the birefringence is only due to the crystals (namely, the spherulites) [22]. In Figure 7, the measured birefringence of all the samples is reported versus the amount of *α* phase of each film. It can be noticed that about all the points collect on the same plot which is essentially linear, independently of the steps of recycling and of the cooling rates. This confirms that in the samples analyzed in this work only the crystalline structures of *α* phase contribute to the birefringence of the samples.

Considering that all the samples have the same surface roughness and thickness, their optical transparency should be mainly related to the crystallinity and since the overall crystallinity, is about the same, the amount of *α* phase could be the controlling factor.

**Figure 8:**
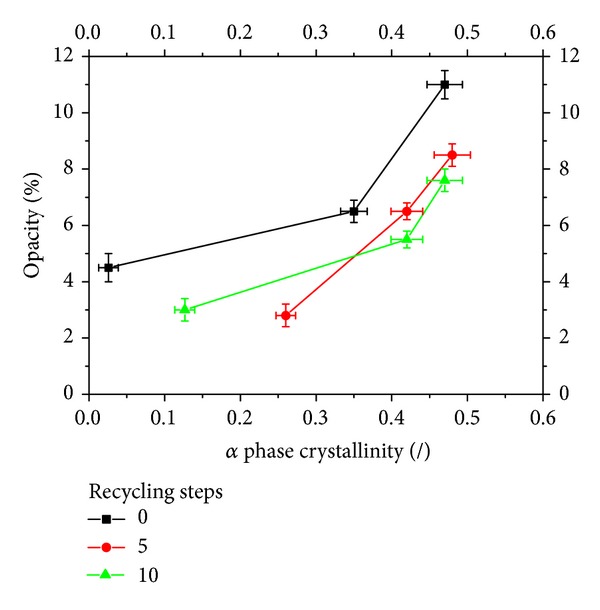
Opacity versus the amount of *α* phase inside each sample.

In Figure 8, opacity is plotted versus the amount of *α* phase inside each sample. It can be noticed that opacity generally increases on increasing the crystallinity degree; however, the effect depends also (in a nonmonotonous way) on the steps of recycling.

**Figure 9:**
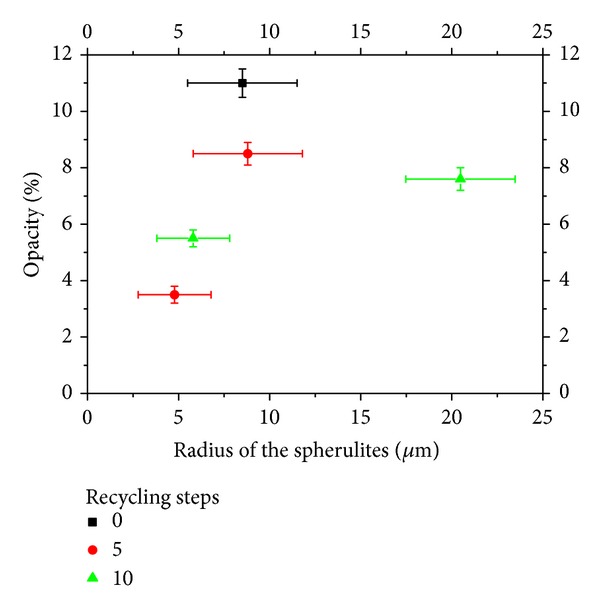
Dependence of the opacity of the films versus the radius of the spherulites for samples presenting an *α* phase content between 0.4 and 0.5.

A further insight into the property can be given by analyzing the opacity of the samples which present the same crystallinity degree. In particular, the samples present an  *α*  phase content between 0.4 and 0.5, regardless of the cooling rate and the recycling steps. Each of those samples presents different average spherulite radii, which can influence the opacity. Thus the opacity is reported in Figure 9 versus the average radius of the spherulites measured on each sample. It can be noticed that opacity, for the same crystalline content, tends to increase on increasing the radius of the spherulites.

## 4. Conclusions

In this work, the influence of recycling steps on the opacity of films of a commercial grade of isotactic polypropylene was studied. The material was extruded several times to mimic the effect of recycling procedures. After extrusion, films were obtained by cooling samples of material at different cooling rates, taking care of the fact that all the samples present the same surface finish. The opacity of the obtained films was then measured and related to their crystallinity and morphology. It was found that opacity generally increases on increasing the amount of *α* phase; however, the effect depends also (in a nonmonotonous way) on the steps of recycling, mainly because the samples underwent different recycling steps, even when they present the same crystallinity degree, and the same amount of *α* phase can have spherulites of different average sizes. It was shown that, for the same amount of *α* phase, opacity generally increases on increasing the size of the spherulites.

## Figures and Tables

**Table 1:** Processing parameters.

Screw speed (rpm)	50
*L*/*D* ratio (—)	24
Die width (mm)	100
Screw diameter (mm)	25
Die gap (mm)	2
Die temperature (°C)	210

**Table 2:** Cooling rates imposed during solidification and average radius of the spherulites.

Recycling steps	Cooling rate at 70°C (K/s)	Radius (*μ*m)
0	0.3	8.5 ± 3
0	5	Not detectable
0	110	n.d.
5	0.3	8.8 ± 3
5	5	4.8 ± 2
5	170	n.d.
10	0.3	20 ± 3
10	5	5.8 ± 2
10	180	n.d.
